# How Affective Polarization Shapes Americans’ Political Beliefs: A Study of Response to the COVID-19 Pandemic

**DOI:** 10.1017/XPS.2020.28

**Published:** 2020-08-24

**Authors:** James N. Druckman, Samara Klar, Yanna Krupnikov, Matthew Levendusky, John Barry Ryan

**Affiliations:** 1 Department of Political Science, Institute for Policy Research, Northwestern University, Evanston, IL, USA; 2 School of Government and Public Policy, University of Arizona, Tucson, AZ, USA; 3 Department of Political Science, Stony Brook University, Stony Brook, NY, USA; 4 Department of Political Science, Annenberg Public Policy Center, University of Pennsylvania, Philadelphia, PA, USA

**Keywords:** Affective polarization, COVID-19, policy opinion, attribution of responsibility, partisanship, experiment

## Abstract

Affective polarization – partisans’ dislike and distrust of those from the other party – has reached historically high levels in the United States. While numerous studies estimate its effect on apolitical outcomes (e.g., dating and economic transactions), we know much less about its effects on political beliefs. We argue that those who exhibit high levels of affective polarization politicize ostensibly apolitical issues and actors. An experiment focused on responses to COVID-19 that relies on pre-pandemic, exogenous measures of affective polarization supports our expectations. Partisans who harbor high levels of animus towards the other party do not differentiate the “United States’” response to COVID-19 from that of the Trump administration. Less affectively polarized partisans, in contrast, do not politicize evaluations of the country’s response. Our results provide evidence of how affective polarization, apart from partisanship itself, shapes substantive beliefs. Affective polarization has political consequences and political beliefs stem, in part, from partisan animus.

## Introduction

A defining feature of 21^st^ century American politics is the rise of affective polarization – the tendency of partisans to dislike, distrust, and avoid interacting with those from the other party (Iyengar, Sood, and Lelkes 2012). Today, such partisan discord has reached record high levels (Pew Research Center 2019) and it affects many apolitical aspects of our lives: for example, where we shop, our friendships, and our romantic lives (for a review, see Iyengar et al. 2019). But how does affective polarization affect our politics? Surprisingly, we do not know much about this relationship: “little has been written on this topic (i.e., the political effects), as most studies have focused on the more surprising apolitical ramifications” (Iyengar et al. 2019, 139). Here, we investigate one aspect of that puzzle: how does affective polarization shape our policy beliefs?

Demonstrating this relationship is fundamental to our understanding of how policy preferences develop, particularly in our present political moment. If affective polarization shapes issue beliefs, it would (1) constitute direct evidence that citizen polarization matters for politics and (2) suggest that policy attitudes stem partially from animus, rather than simply from more substantive rationales (cf. Fowler 2020). The scarcity of work documenting such an effect, however, reflects the extreme difficulty of doing so. Issue positions are endogenous to partisan animus: elite polarization drives both affective polarization (Rogowski and Sutherland 2016; Webster and Abramowitz 2017), as well as issue positions (via cue-taking, see Lenz 2012). Unsurprisingly, those who are more affectively polarized tend to also hold more polarized issue positions (e.g., Bougher 2017), so it is unclear whether the relationship between issue positions and affective polarization is a causal one or rather a product of other factors that jointly lead to both outcomes.^1^

To unpack these effects, one would need a measure of affective polarization taken prior to the emergence of an issue, something that is impossible to predict and thus difficult to accomplish. The COVID-19 pandemic, however, presents us with a means of doing so. Because the virus and resulting pandemic was completely novel when it emerged in early 2020, partisans did not have prior beliefs about it and their pre-COVID levels of affective polarization cannot be affected by how elites acted during the crisis. A pre-COVID measure of affective polarization, therefore, allows us to determine the relationship between partisan animus and beliefs about the pandemic. This not only enables us to uniquely isolate whether affective polarization shapes policy attitudes, but it also provides essential insight into the COVID-19 crisis. If affective polarization divides the public, it creates hurdles for policymakers as they develop strategies to combat the pandemic now and in the future. It is not simply that there are partisan divides on the severity and handling of the crisis (e.g., Gadarian, Goodman, and Pepinsky 2020, and McCarthy 2020), but rather that dislike of the opposition, at least in part, drives such gaps. This implies that policymakers and communicators must not only find substantive policies that bridge differing partisan priorities, but they also must find a way to vitiate partisan animus, a much more difficult task.

## How does affective polarization shape responses to the crisis?

A long line of political science research suggests that partisanship shapes how people interpret the political world (Bartels 2002), and how they assess credit and blame for governments’ responses to crises (Malhotra and Kuo 2008). The COVID-19 pandemic has been no exception, with surveys highlighting large partisan gaps in the perceived seriousness of the crisis, actions taken in response to it, and assessments of blame for the outcome (Allcott et al. 2020; Gadarian et al. 2020). Much like other policies, even health pandemics have become partisan issues in the contemporary USA.

At first blush, it might seem clear that partisan animus would lead to clear divides on political issues. Yet, as we noted above, simply because partisans take different positions on issues does *not* mean that these positions are a function of affective polarization: for example, partisans might hold differential factual beliefs about the world (Gerber and Green 1999; Fowler 2020) or have different underlying values (Goren 2005). In the case of COVID-19, Republicans might see different information about the pandemic, or they might value economic stability more than Democrats do, both of which would lead to partisan differences even in the absence of animus. Given the existing evidence, we cannot conclude that affective polarization drives partisan differences in response to the pandemic.

But there is reason to think that affective polarization, apart from partisan identification itself, can influence individuals’ policy beliefs. Specifically, affective polarization, perhaps ironically, will not affect *politicized* aspects of the issue. Rather, political divisions in these areas manifest regardless of the level of polarization. When issues are already politicized, even those with low levels of affective polarization see them through a partisan lens. Affective polarization rather politicizes ostensibly neutral targets, leading affectively polarized individuals to see apolitical topics through the prism of partisanship.

We focus here on how Americans evaluate the country’s national COVID-19 response. A unified response to this pandemic is central to ensuring collective success in defeating it. If affective polarization divides Democrats from Republicans, then it becomes more difficult to move forward with a coherent policy to address the crisis. Prior work on attributions shows that partisan labels shape evaluations of government actors: individuals express greater confidence in, and more positive evaluations of, co-partisans (e.g., Malhotra and Kuo 2008; Healy et al. 2014). This should straightforwardly apply to COVID-19. Here, we compare beliefs about “President Trump’s” response to the pandemic to beliefs about the “United States’” response to it. The former clearly invokes a highly politicized (and polarizing) individual. The latter is a more neutral entity; also, using the nation as a whole primes national identity, which should mute the effects of partisanship (Levendusky 2018). Further, evaluations of how one’s country is handling the crisis are important as they tell us about cross-national assessments of governmental response to COVID-19 (Dryhurst et al. 2020). While we expect there to be a partisan split in response to President Trump’s handling of the crisis, it should not be driven by affective polarization, as all citizens will divide along party lines in response to such a politicized figure. Asking about the country, however, need not evoke a partisan response – there is no reason for Democrats overall to evaluate the United States’ response poorly whereas there is a clear partisan reason for them to evaluate Trump’s response poorly (and similarly for Republicans in terms of no need to politicize the US response). This leads to our first hypothesis.

H1: *Democrats (Republicans) will be less (more) critical of the United States’ response to COVID-19, relative to Trump’s response to COVID-19, all else constant.*

We expect that affectively polarized partisans will politicize references to the country, seeing the national response through a partisan lens. This will lead them to equate the “United States” with the federal government – and hence President Trump – similarly to how affectively polarized citizens politicize trust in the government as a whole (Hetherington and Rudolph 2015). For affectively polarized individuals, partisanship is chronically accessible and shapes their views of ostensibly neutral, or even potentially unifying, targets. They will see the “United States” as synonymous with, or at least similar to, “President Trump,” thereby politicizing it. They want to signal their partisan identity whenever possible (to make sure to distinguish themselves from the other side).

H2: *As affective polarization increases, Democrats (Republicans) will be more (less) critical of the United States’ response to COVID-19, all else constant.*

A consequence of H2 is that the treatment effect predicted in H1 will decrease or disappear among affectively polarized individuals since they view all targets politically (*corollary 1*). In short, corollary 1 follows logically from H1 and H2 (i.e., we expect a treatment effect that will shrink as affective polarization increases). Our hypotheses concern Democrats and Republicans and, as such, do not apply to pure Independents; we thus follow other work on affective polarization and exclude pure Independents from our analyses (Druckman and Levendusky 2019). We pre-registered this exclusion and our hypotheses prior to the completion of data collection – specifically stating the hypotheses in terms of corollary 1 (for Democrats and Republicans) – at: https://aspredicted.org/7pd2i.pdf. A copy of our stage 1 manuscript written prior to the data analysis is available at the Dataverse link: https://doi.org/10.7910/DVN/SLDUUT.

We also test an alternative hypothesis, suggested by a reviewer but not initially pre-registered, that a measure of partisanship as a social identity moderates the treatment effect. Partisanship as a social identity is a construct that captures the extent to which one feels as if he or she belongs to the party (e.g., when talking about the party, how often does the individual use “we” instead of “they,” see Huddy, Mason, and Aarøe 2015). While a plausible alternative, we did not pre-register this idea as our theory focuses on the out-party animus nature of affective polarization (which partisan social identity lacks). That negativity, we suspect, drives partisans to politicize issues to signal their partisan identities and differentiate themselves from the other side they dislike. We present these results here in the interest of transparency.

## Experimental design

Issues of endogeneity make it difficult to determine whether affective polarization shapes responses to COVID-19 or any other issue. A correlation between contemporaneous affective polarization and COVID-19 opinions could stem from polarization causing beliefs about COVID-19, or from elite debates about COVID-19 heightening affective polarization. We need data that measure affective polarization *before* people form issue opinions – in this case, prior to the outbreak of COVID-19.

To circumvent this problem, we rely on a survey of a representative sample of 3,345 participants conducted in the summer of 2019 (from July 9, 2019 to July 25, 2019), prior to the emergence of COVID-19 as an issue. In our pre-registration document, we stated that our initial sample included more than 4,000 individuals; however, prior to launching data collection, we realized that numerous respondents had not answered relevant affective polarization measures in our initial survey. As a result, the relevant re-contact sampling frame (who had answered the key affective polarization measure) was 3,345. Hence, there is a discrepancy here with the pre-registration sampling frame number (see Supplementary Information [SI] 1 for more details on this original study).

The summer 2019 survey included four canonical measures of affective polarization (Druckman and Levendusky 2019): feeling thermometer ratings toward the parties (i.e., a scale where 0 indicates very cold feelings and 100 indicates very warm feelings), the degree to which respondents trust out-partisans versus in-partisans, trait ratings of opposing partisans (i.e., asking how well adjectives like patriotic, open-minded, etc. apply to out-partisans), and social distance measures that ask people how comfortable they would be to have a friend or neighbor from the other party, or how happy they would be if they had a child who married someone from the other party. We aggregate these items to form a measure of affective polarization (*α* = 0.88), looking specifically at out-party animus (e.g., Lau et al. 2017). We scale this measure to lie between 0 and 1, with higher values indicating greater animosity for the other party. Due to the timing of our measure of affective polarization, we can be confident that it is unrelated to the politics surrounding COVID-19, thereby allowing us to draw causal inferences about its effects on COVID-19 beliefs. The summer 2019 survey also included a four-item scale to measure partisanship as a social identity – including questions about the importance of one’s party, how well the party label describes the individual, the use of the word “we” when thinking of the party, and the extent one thinks of him/herself as being in that party. This scaled measure (*α* = 0.90) enables us to test the aforementioned alternative hypothesis put forth by a reviewer.

We re-interviewed these same respondents in the spring of 2020 (from April 4, 2020 to April 16, 2020), measuring their assessments of the handling of the COVID-19 crisis to isolate the causal impact of affective polarization. A total of 2,484 participants completed the re-interview for a re-contact rate of 74% (see SI 1 for more details on the sample demographics). The re-interview survey included one measure of affective polarization – the feeling thermometer item – and we find, consistent with prior work (Alwin 1997; Beam et al. 2018), that it is relatively stable over time: there is a correlation of .76 between the original and re-interview out-party thermometer evaluation. This gives us confidence that the affective polarization measures from the pre-COVID-19 surveys serve as valid and reliable measures of exogenous affective polarization.

The COVID-19 survey included an experiment to test our hypotheses. Specifically, we randomly assigned participants to one of two conditions where they assessed the response to the COVID-19 pandemic. One group was asked about President Trump’s response, while the other was asked about the United States’ response. In each condition, we measured assessments on three items: (1) confidence to address the pandemic (e.g., how confident are you that the Trump administration/United States can limit the impact of the virus), (2) response to the past preparation for the current outbreak (i.e., disagreement or agreement that President Trump/the United States should have done more to prepare for the outbreak), and (3) preparation for potential future outbreaks (i.e., disagreement or agreement that President Trump/the United States should be doing more to prepare for the possibility of a future outbreak).

If the results are consistent with our hypotheses, we should observe the following pattern of results. First, in line with Hypothesis 1, we would observe that participants from different political parties offer differential evaluations of the targets (e.g., Republicans being more favorable about Trump than the United States). Next, we expect to see that affective polarization moderates this relationship with a significant interaction between the US treatment and affective polarization (Hypothesis 2). Finally, we also expect that affectively polarized individuals do not differentiate in their assessments of President Trump and the United States, meaning that we may not observe any treatment effects among those who are most affectively polarized (corollary 1). In short, we expect that those who are not affectively polarized will differentiate evaluations of President Trump and the United States – viewing the superordinate category of the United States as something distinct from partisanship. In contrast, those who are more affectively polarized will politicize that superordinate construct, creating a divide even on an ostensibly apolitical target. The questionnaire for both surveys is provided in SI 2.

## Results

Once we exclude pure Independents, as explained above and in accordance with prior work, our dataset includes 2,124 partisans.^2^ We create a scale (ranging from 1–4, with higher values indicating more approval/confidence) from our three evaluation measures (*α* = .76; see SI 3 for results presented separately for each measure).^3^ To test the first hypothesis, we run a model that includes only a variable for treatment assignment (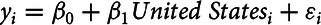), where 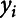 is respondent *i*’s attitude about the response to the pandemic and 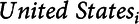 is an indicator for whether respondent *i* was asked about the United States’ handling of the crisis (versus Trump’s). To test our second hypothesis, as well as the corollary, we run the following regression: 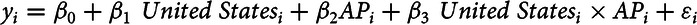, where the additional variable, 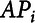, is the participant’s level of affective polarization (measured in 2019).^4^

In Table 1, we present the results separately for Democrats and Republicans, as we have separate expectations for the parties. We begin with the Democrats and turn first to the test of Hypothesis 1 (Table 1, Model 1). We see that Democrats offer more favorable evaluations of America’s response to COVID-19 when asked about the United States’ response relative to Trump’s response (difference of 0.25, *p* < 0.01 for a one-tailed test). This follows from Hypothesis 1: when asked about the response in the context of the United States, rather than the President, Democrats are overall more positive.^5^

**Table 1:** Evaluations by Party by Experimental Condition

	Democrats		Republicans	
	Model 1	Model 2	Model 3	Model 4
US condition	0.254**	0.463**	−0.307**	−0.626**
	(0.033)	(0.100)	(0.055)	(0.167)
Aff. pol.		−0.903**		1.208**
		(0.115)		(0.214)
US X Aff. pol.		−0.359*		0.592*
		(0.164)		(0.301)
Constant	1.438**	1.957**	2.569**	1.936**
	(0.023)	(0.070)	(0.040)	(0.118)
Observations	1,389	1,389	734	734
*R*-squared	0.042	0.151	0.040	0.160

*Notes:* Standard errors in parentheses.

Aff. pol.; affective polarization.

** *p* < 0.01, * *p* < 0.05, for one-tailed tests.

We next turn to our test of Hypothesis 2 (Table 1, Model 2). Here, we see a significant interaction between affective polarization and treatment assignment. Turning to the substantive effects of this interaction, we see outcomes that are consistent with our predictions. First, increases in affective polarization among Democrats have a significant, negative effect on evaluations of the response to COVID-19 in *both* conditions. When participants are asked about the United States, increases in affective polarization lower evaluations of the country’s response by –1.262 (*p* < 0.01); when participants are asked about Trump, increases in affective polarization lower evaluations by -0.903 (*p* < 0.01).^6^ This is in line with Hypothesis 2, which posits that as affective polarization increases, Democrats will become more negative toward the American response.

The results for Republicans are nearly identical but in the opposite direction, as expected. First (Table 1, Model 3), Republicans exhibit a lower evaluation of America’s response to COVID-19 when the target is the United States as opposed to Trump (–0.31, *p* < 0.01). This result is in line with Hypothesis 1.^7^ Next, we again see a significant interaction between affective polarization and treatment in Table 1, Model 4. Following Hypothesis 2, as affective polarization increases, Republicans become less critical of the American response in the United States (1.800, *p < 0.001*); they also become less critical of Trump response (1.208, *p < 0.01*).^8^

**Figure 1:**
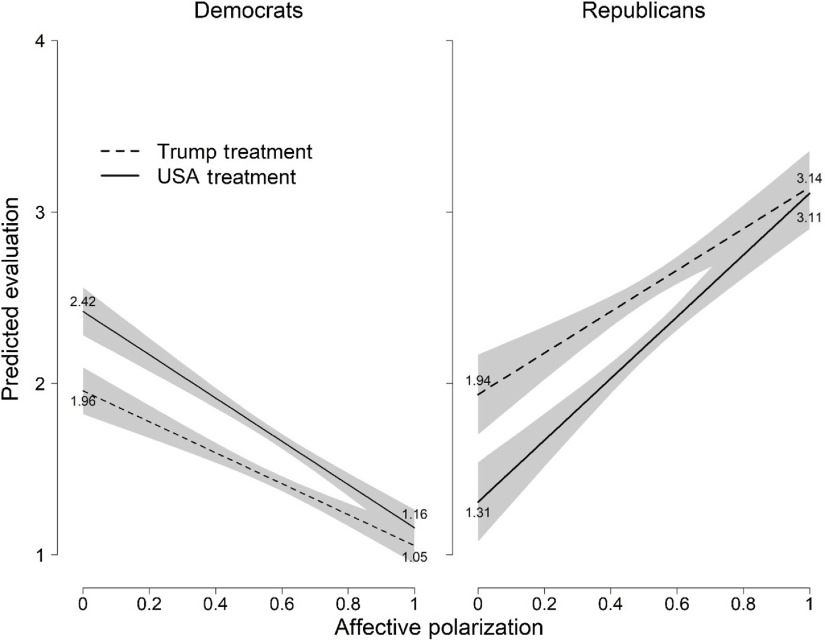
Predicted Evaluations from Table 1’s Model 2 for Democrats and Table 1’s Model 4 for Republicans. *Notes*: Shaded area represents 95% confidence interval.

We next consider another set of results suggested by corollary 1, which we present in Figure 1. In this figure, we plot the predicted values for each party, for each experimental condition at different levels of affective polarization. In the United States treatment, Democrats with low levels of polarization evaluate America’s response to COVID-19 at 2.42, substantially surpassing the evaluations in the Trump treatment (1.96). This difference between treatments is significant allowing us to reject the null hypothesis of no difference (+0.46, *p* < 0.01). Yet, the Democratic lines converge as polarization increases such that at the highest level of polarization, the United States and Trump scores are extremely similar (respectively at 1.16 and 1.05) and we cannot reject the null hypothesis of no difference (+0.103, *p* = .087). In sum, highly polarized Democrats’ evaluations of “the United States” response are not statistically different from their evaluations of the “Trump” response. In both cases, they politicize the potentially superordinate target.

We see similar dynamics among Republicans. Republicans with low levels of affective polarization report higher evaluations of the American response in the Trump condition than in the United States condition such that we can reject the null hypothesis of no difference (1.94 versus 1.31, difference of −0.626, *p* < 0.01). Yet the evaluations of the targets converge for Republicans who are high in affective polarization and we cannot reject the null hypothesis of no difference (respectively, to 3.14 and 3.11, difference of −0.035, and *p* = 0.410).^9^ The figure makes clear that affective polarization has a causal impact on political assessments, leading partisans to politicize evaluations even in cases with an, ostensibly, neutral target. This is concerning insofar as affective polarization leads partisans to split when evaluating the country overall, undermining confidence in the national response which ideally would connect all citizens.

Finally, we turn to the aforementioned alternative hypothesis, offered by a reviewer, that the moderator is partisanship as a social identity rather than affective polarization. To test this, we run the interactive models from Table 1 but instead of our affective polarization measure, we include a partisanship as a social identity measure. We provide these results in Table 2.

**Table 2:** Results with Partisanship as a Social Identity

	Democrats Model 5	Republicans Model 6
US condition	0.367**	−0.157
	(0.107)	(0.176)
Social identity	−0.031	0.189**
	(0.023)	(0.041)
US X identity	−0.036	−0.051
	(0.032)	(0.058)
Constant	1.539**	2.022**
	(0.076)	(0.124)
Observations	1,388	734
*R*-squared	0.050	0.082

*Notes:* Standard errors in parentheses.

** *p* < 0.01, * *p* < 0.05 for one-tailed tests.

We find the results do not replicate with that construct, suggesting that it is affective polarization generating our effects (as we pre-registered). The distinct results likely stem from the fact that partisanship as a social identity does not have an out-party animus component, which is what politicizes beliefs – pushing individuals to want to affirm their partisan identity and reject the other side.

## Conclusion

The rise in affective polarization has captured the attention of scholars, pundits, and citizens, yet we know little about its political effects and especially its effect on political issues. Our study is the first to use a clearly exogenous measure of affective polarization to show how partisan animus shapes respondents’ beliefs about a political issue. Specifically, we show that affective polarization has little effect on already politicized issues, but it politicizes ostensibly neutral or apolitical ones. This makes clear that affective polarization or “political tribalism” is much more than mere reflections of policy preferences (Fowler 2020). It also highlights the reciprocal relationship between affective and ideological polarization, and it suggests that the two are quite intimately linked.

Our study also has implications for the ongoing response to the COVID-19 pandemic. Even ostensibly neutral communications become politicized by those who are highly polarized, thereby necessitating additional techniques to de-polarize them (e.g., bi-partisan endorsements; see Bolsen et al. 2014). In particular, it suggests that superordinate appeals to the nation (Van Bavel et al. 2020) are ineffective for those who are most polarized, and hence policymakers need to craft strategies to appeal directly to them and work on de-polarization strategies rather than appeals to a shared identity.

Beyond this particular pandemic, our results speak more to the power of affective polarization to politicize novel issues and ongoing political debates. Partisans who are more affectively polarized – who are also more politically engaged – politicize neutral issues and will polarize on most topics with only weak elite cues. Our findings constitute the first evidence that affective polarization has clear policy implications as it divides opinion on those political issues that appear non-partisan or even apolitical. It highlights the importance of efforts to de-polarize partisans, as it may be the only route to coherent policy agendas.

## Footnotes

1 It also is extremely difficult to experimentally manipulate levels of affective polarization due to extensive pre-treatment and ceiling effects among the more politically engaged segments of the public (see Pew Research Center 2019).

2 We note, however, that one respondent did not answer any of our main outcome measures.

3 The items also scale well if we look at the experimental conditions separately (α = .80 for the Trump condition and α = .71 for the United States condition), or at the parties separately (α = .68 for Democrats and α = .68 Republicans).

4 Our pre-registration stated that we would only run the second model, which contains the interaction between affective polarization and treatment. At the suggestion of a reviewer on the initial pre-registered report, we present both models as it provides a direct test of the treatment not conditioned by affective polarization.

5 As per our prior note, we see in Table 1, Model 2, the treatment variable is significant as is the coefficient on the interaction term. An analysis of the marginal effect of the treatment shows that the treatment has a statistically significant effect at the .05 level in a one-tailed test when the affective polarization variable ranges from 0 to about .95.

6 The effects of increasing polarization by treatment have overlapping confidence intervals, suggesting they are likely not statistically distinguishable from each other.

7 Again, we can see support for hypothesis 1 with the significant effect of the treatment variable in model 4.

8 The effects of increasing polarization by treatment have overlapping confidence intervals, suggesting they are not statistically distinguishable from each other. In SI 3, we present some exploratory analyses these show the results are robust to the inclusion of a host of control variables.

9 One intriguing exploratory finding is the least polarized Democrats evaluate the response in the Trump condition at virtually the same level as the least polarized Republicans, perhaps reflecting a low levels of partisan reasoning. Also, the least polarized Republicans have much less favorable evaluations of the United States than the least polarized Democrats

## Supplementary material

For supplementary material accompanying this paper visit http://dx.doi.org/10.1017/XPS.2020.28. click here to view supplementary material
