# Supplementary material for: How Affective Polarization Shapes Americans’ Political Beliefs: A Study of Response to the COVID-19 Pandemic
Source: Journal of Experimental Political Science. 2020 Aug 24:1–12. doi: 10.1017/XPS.2020.28 (PMC7550884; doi:10.1017/XPS.2020.28)
Supplement: Supplementary file 1 [file S2052263020000287sup001.docx]

**Supplementary Information for How Affective Polarization Shapes Americans’ Political Beliefs: A Study of Response to the COVID-19 Pandemic**

**Supplementary Information 1: Sample**

The survey was conducted using Bovitz Inc. (<http://bovitzinc.com/index.php>). They provide an online panel of approximately one million respondents recruited through random digit dialing and empanelment of those with internet access. As with most internet survey samples, respondents participate in multiple surveys over time and receive compensation for their participation. A particular sample is drawn using a matching algorithm (based on likely response rates) to ensure that those screened to qualify for the survey constitute an unweighted sample that demographically represents the United States. Bovitz Inc. has been used extensively in other political science research (e.g., Howat 2019, Druckman and Levendusky 2019) including pilot data collection for the American National Election Studies.

The initial survey that included basic demographics and our affective polarization measures took from 9 July 2019 to 25 July 2019. The total number who completed the survey, answering the affective polarization measures was 3,345 (for the discrepancy mentioned in the text between this N and the one pre-registered see the accompanying footnote).^[[1]](#footnote-1)^ We re-contacted these individuals in April, 2020 to answer the COVID-19 items. A total of 2,484 responded, for a re-contact rate of 74%. Of these, 360 are pure Independents and thus excluded from our main analyses, as noted. This leaves 2,124 partisans for analyses, although one respondent did not answer any of the experimental outcome variables, leaving 2,123 for analysis.

The below tables present the demographics of our COVID-19 sample to 2018 benchmarks from the U.S. Census Bureau, via the American Community Survey.^[[2]](#footnote-2)^

Age

| Age Category | Our Sample (%) | Census Benchmark |
| --- | --- | --- |
| 18-24 | 8.29 | 12.08 |
| 25-34 | 18.40 | 17.87 |
| 35-50 | 35.39 | 24.54 |
| 51-65 | 26.61 | 24.88 |
| Over 65 | 11.31 | 20.65 |

Gender Identity

| Gender Identity | Our Sample (%) | Census Benchmark |
| --- | --- | --- |
| Female | 51.03 | 50.8 |
| Male | 48.09 | 49.2 |
| Transgender/None | .88 | --^[[3]](#footnote-3)^ |

Primary Racial Group*

| Primary Race | Our Sample (%) | Census Benchmark |
| --- | --- | --- |
| Caucasian (White) | 70.73 | 72.2 |
| African-American | 13.81 | 12.7 |
| Hispanic or Latino | 9.02 | 18.3 |
| Asian-American | 3.99 | 5.6 |
| Native American | .85 | < 1 |
| Other | 1.61 | 5 |

*The Census asks about ethnicity (Hispanic/Latino) separately from race, whereas we combine them into one question and ask respondents to report their “primary” group. As a result, our estimates for Hispanic/Latino citizens are measuring a different construct from the Census benchmark.

Annual Family Income before Taxes*

| Income Category | Our Sample (%) | Census Benchmark (%) |
| --- | --- | --- |
| $30,000 or less | 26.83 | 29.4 |
| $30,000 - $69,999 | 38.11 | 30.3 |
| $70,000 - $99,999 | 17.37 | 12.5 |
| $100,000 - $200,000 | 15.27 | 20.9 |
| Above $200,000 | 2.42 | 6.9 |

* The Census categories for income are slightly different than the ones we use. They record income as: $34,999 or below, $35,00 - $74,999, $75,000 - $99,999, $100,000 - $199,999, and $200,0000 or greater.

Education Level

| Educational Attainment | Our Sample (%) | Census Benchmark (%) |
| --- | --- | --- |
| Did not complete high school | 2.01 | 12 |
| High school graduate | 20.41 | 27.1 |
| Associates Degree/Some College | 41.18 | 28.9 |
| Bachelor’s Degree | 26.69 | 19.7 |
| Advanced Degree | 9.70 | 12.3 |

Across categories, our sample matches the Census benchmarks fairly well. Our biggest discrepancies are that (1) we under-estimate senior citizens and over-estimate 35-50 year olds, (2) we possibly under-estimate Latinos (although that may stem from our question format, as noted), (3) we under-estimate the top quarter of the income distribute, and (4) we under-estimate the least well-educated (and over-estimate those with some college or a bachelor’s degree). These are well-known limitations of any survey sampling procedure, not just our own—problems #1 and #3 are linked in that those populations are not online, and those with high incomes are also typically under-represented across all survey modes. Overall, however, our sample sufficiently matches the Census benchmarks across these different categories for the purposes of our experiment (in which we have no expectation of moderating effects of demographics).

**Supplementary Information 2: Measures**

2019 Items:^[[4]](#footnote-4)^

We are going to ask you some questions about your general attitudes and opinions.

Generally speaking, do you usually think of yourself as a Democrat, a Republican, an Independent, or what?

*Democrat Republican Independent Some other party*

**[IF D/R:]**

Would you call yourself a strong [**Democrat** / **Republican**] or a not very strong [**Democrat** / **Republican**]?

*Strong Not very strong*

**[IF I/O:]**

If you had to choose, do you think of yourself as closer to the Democratic Party or the Republican Party?

*Closer to Closer to Neither*

*Democratic Party Republican Party*

Which point on the scale below best describes your political views?

*Very Mostly Somewhat Moderate Somewhat Mostly Very*

*liberal liberal liberal conservative conservative conservative*

What is the highest level of education you have completed?

*Less than High Some 4 year college Advanced*

*High school school graduate college degree degree*

What is your estimate of your family’s annual household income (before taxes)?

*< $30,000 $30,000 - $69,999 $70,000-$99,999 $100,000-$200,000 >$200,000*

Which of the following do you consider to be your primary racial or ethnic group?

*White African American Asian American Hispanic or Latino Native American Other*

Which of the following best describes your gender identity?

*Male Female Transgender None of the categories offered*

What is your age?

*Under 18 18-24 25-34 35-50 51-65 Over 65*

*Many people don’t know the answers to these questions, so if there are any you don’t know, just check “don’t know.”*

How much of a majority is required for the U.S. Senate and House to override a Presidential veto?

*Cannot 1/3 1/2 2/3 3/4 Don’t know*

*override*

Do you happen to know which party currently has the most members in the House of Representatives in Washington, D.C.?

*Democrats Republicans Tie Don’t know*

Whose responsibility is it to determine if a law is constitutional?

*President Congress Supreme Court Don’t know*

Who is the current U.S. Vice President?

*Rex Tillerson James Mattis Mike Pence Paul Ryan Don’t know*

Would you say that one of the major parties is more conservative than the other at the national level? If so, which party is more conservative?

*The* *Democratic Party The Republican Party Neither Don’t know*

*We are now going to ask a few more questions about your partisanship.*^[[5]](#footnote-5)^

How important is being a $PARTY to you?

*Not at all Not very Somewhat Very Extremely*

*important important important important important*

How well does the term $PARTY describe you?

*Not at all Not very Somewhat Very Extremely*

*well well well well well*

When talking about $PARTYs, how often do you use “we” instead of “they”?

*Never Rarely Some of Most of All of*

*the time the time the time*

To what extent do you think of yourself as being a $PARTY?

*Not at all Not too much Somewhat A good deal A great deal*

We’d like you to rate how you feel towards $OUTGROUP on a scale of 0 to 100, which we call a “feeling thermometer.” On this feeling thermometer scale, ratings between 0 and 49 degrees mean that you feel unfavorable and cold (with 0 being the most unfavorable/coldest). Ratings between 51 and 100 degrees mean that you feel favorable and warm (with 100 being the most favorable/warmest). A rating of 50 means you have no feelings one way or the other. How would you rate your feeling toward these groups? Remember we are asking you to rate ordinary people (e.g., voters) and *not* elected officials, candidates, media personalities, etc.

$OUTGROUP^[[6]](#footnote-6)^

We’d like to know more about what you think about $OUTGROUP. Below, we’ve given a list of words that some people might use to describe them.

For each item, please indicate how well you think it applies to $OUTGROUP: not at all well; not too well; somewhat well; very well; or extremely well.^[[7]](#footnote-7)^

|  | Not at all well | Not too well | Somewhat well | Very well | Extremely well |
| --- | --- | --- | --- | --- | --- |
| Patriotic |  |  |  |  |  |
| Intelligent |  |  |  |  |  |
| Honest |  |  |  |  |  |
| Open-minded |  |  |  |  |  |
| Generous |  |  |  |  |  |
| Hypocritical |  |  |  |  |  |
| Selfish |  |  |  |  |  |
| Mean |  |  |  |  |  |

How much of the time do you think you can trust $OUTGROUP to do what is right for the country?

*Almost Once in a About half Most of the Almost*

*never while the time time always*

How comfortable are you having close personal friends who are $OUTGROUP?

*Not at all Not too Somewhat Extremely*

*comfortable comfortable comfortable comfortable*

How comfortable are you having neighbors on your street who are $OUTGROUP?

*Not at all Not too Somewhat Extremely*

*comfortable comfortable comfortable comfortable*

Suppose a son or daughter of yours was getting married. How would you feel if he or she married someone who is a $OUTGROUP?^[[8]](#footnote-8)^

*Not at all Not too Somewhat Extremely*

*upset upset upset upset*

2020 Wave:

[RANDOMLY ASSIGN SUBJECTS TO SEE EITHER “THE TRUMP ADMINISTRATION” OR “THE UNITED STATES” BELOW. TREATMENT ASSIGNMENT IS HELD CONSTANT ACROSS ITEMS, SO SUBJECTS SEE THE SAME WORDING IN ALL ITEMS.]

How confident are you that the [Trump administration/United States] can limit the impact of the coronavirus in the next month?^[[9]](#footnote-9)^

1. Not confident at all
2. A little confident
3. Pretty confident
4. Very confident

Do you disagree or agree that [President Trump/the United States] should have done more to prepare for the coronavirus outbreak we are currently experiencing?

1. Strongly disagree
2. Somewhat disagree
3. Somewhat agree
4. Strongly agree

Do you disagree or agree that [President Trump/the United States] should be doing more right now to prepare for the possibility of a new outbreak of the coronavirus in the fall?

1. Strongly disagree
2. Somewhat disagree
3. Somewhat agree
4. Strongly agree

How often have you relied Fox News for information about the coronavirus outbreak?

1. Never
2. Rarely
3. Sometimes
4. Frequently
5. Every Day

Does any of your work currently require you to leave home?

1. No
2. Yes, for a little of my work.
3. Yes, for some of work.
4. Yes, for all my work.

Do you, or does anyone in your household, work in health care?

1. No
2. Yes

Do you have children under 4 years old living with you?

1. No
2. Yes

Are you or your spouse currently pregnant?

1. No
2. Yes

Do you currently have any health conditions that would make the coronavirus especially risk for you, such as asthma, emphysema, or difficulty breathing?

1. No
2. Yes

**We obtained each respondent’s county of residence from the data vendor to match for COVID-19 case data (per capita) from The New York Times.*

**Supplementary Information 3: Additional Analyses**

In Tables S3-1-2, we present the results for each outcome variable. These were not pre-registered by we believe worth exploratory analyses. The results show that the results are not entirely consistent across outcome variables when broken down in terms of statistical significance, but always in the correct direction. Specifically, for Democrats, the statistical results replicate for past preparation and future preparation, but the interaction falls short of significance for confidence. For Republicans, the statistical results replicate for confidence but the interaction falls short of significance for past preparation and future preparation.

In Table S3-3, we replicate the main results with control variables, showing they are robust.

**Table S3-1: Results for Each Outcome Variable**

|  | (1) | (2) | (3) | (4) | (5) | (6) |
| --- | --- | --- | --- | --- | --- | --- |
|  | Democrats  Confidence | Republicans Confidence | Democrats  Preparation | Republicans Preparation | Democrats  Future | Republicans Future |
|  |  |  |  |  |  |  |
| U.S. Condition | 0.553** | -0.134* | 0.123** | -0.395** | 0.087* | -0.393** |
|  | (0.044) | (0.065) | (0.040) | (0.074) | (0.039) | (0.073) |
| Constant | 1.555** | 2.661** | 1.347** | 2.558** | 1.410** | 2.489** |
|  | (0.032) | (0.046) | (0.029) | (0.053) | (0.028) | (0.052) |
|  |  |  |  |  |  |  |
| Observations | 1,389 | 734 | 1,389 | 734 | 1,389 | 734 |
| R-squared | 0.101 | 0.006 | 0.007 | 0.037 | 0.003 | 0.038 |

Standard errors in parentheses

** p<0.01, * p<0.05 for one-tailed tests

**Table S3-2: Results for Each Outcome Variable (Interaction)**

|  | (1) | (2) | (3) | (4) | (5) | (6) |
| --- | --- | --- | --- | --- | --- | --- |
|  | Democrats Confidence | Republicans Confidence | Democrats  Past | Republicans  Past | Democrats  Future | Republicans  Future |
|  |  |  |  |  |  |  |
| U.S. Condition | 0.650** | -0.838** | 0.449** | -0.538* | 0.290* | -0.503* |
|  | (0.138) | (0.200) | (0.126) | (0.230) | (0.124) | (0.226) |
| Aff. Pol. | -1.196** | 0.723** | -0.789** | 1.505** | -0.724** | 1.397** |
|  | (0.159) | (0.256) | (0.145) | (0.296) | (0.143) | (0.290) |
| U.S. X | -0.165 | 1.323** | -0.563** | 0.256 | -0.350* | 0.195 |
| Aff. Pol. | (0.227) | (0.360) | (0.208) | (0.416) | (0.205) | (0.407) |
| Constant | 2.244** | 2.282** | 1.802** | 1.769** | 1.826** | 1.756** |
|  | (0.096) | (0.141) | (0.088) | (0.163) | (0.087) | (0.160) |
| Observations | 1,389 | 734 | 1,389 | 734 | 1,389 | 734 |
| R-squared | 0.176 | 0.097 | 0.081 | 0.113 | 0.057 | 0.105 |

Standard errors in parentheses

** p<0.01, * p<0.05 for one-tailed tests

**Table S3-3: Results With Control Variables**

|  | (1) | (2) | (3) | (4) |
| --- | --- | --- | --- | --- |
|  | Democrats | Democrats | Republicans | Republicans |
|  |  |  |  |  |
| U.S. Condition | 0.253** | 0.451** | -0.310** | -0.638** |
|  | (0.031) | (0.095) | (0.053) | (0.166) |
| Aff. Pol. |  | -0.760** |  | 0.899** |
|  |  | (0.112) |  | (0.216) |
| U.S. $\times$ Aff. Pol. |  | -0.342* |  | 0.607* |
|  |  | (0.156) |  | (0.300) |
| County Cases Per | 4.704 | 2.410 | -37.404** | -33.673** |
| Capita | (6.415) | (6.124) | (12.726) | (12.244) |
| Health Vulnerability | -0.014 | -0.014 | -0.058 | -0.026 |
|  | (0.032) | (0.031) | (0.056) | (0.054) |
| Work Out of Home | -0.016 | -0.021 | 0.018 | 0.000 |
|  | (0.036) | (0.034) | (0.059) | (0.057) |
| African-American | -0.041 | 0.008 | -0.024 | -0.091 |
|  | (0.041) | (0.039) | (0.148) | (0.142) |
| Latino | 0.008 | 0.036 | 0.034 | 0.082 |
|  | (0.053) | (0.051) | (0.113) | (0.109) |
| Asian-American | -0.057 | -0.049 | -0.410** | -0.366** |
|  | (0.074) | (0.071) | (0.148) | (0.142) |
| Female | -0.046 | -0.054* | 0.028 | 0.006 |
|  | (0.032) | (0.030) | (0.056) | (0.054) |
| Conservative | 0.058** | 0.032** | 0.154** | 0.106** |
|  | (0.013) | (0.012) | (0.022) | (0.022) |
| Age | -0.046** | -0.048** | 0.050* | 0.046* |
|  | (0.015) | (0.014) | (0.027) | (0.026) |
| Education | -0.039* | -0.035* | -0.013 | 0.005 |
|  | (0.019) | (0.018) | (0.032) | (0.031) |
| Political Knowledge | -0.084** | -0.084** | 0.010 | 0.021 |
|  | (0.012) | (0.012) | (0.023) | (0.022) |
| Income | 0.015 | 0.005 | -0.001 | 0.013 |
|  | (0.016) | (0.015) | (0.028) | (0.027) |
| Watch Fox | -0.165** | -0.111** | -0.258** | -0.222** |
|  | (0.035) | (0.033) | (0.055) | (0.053) |
| Constant | 1.988** | 2.473** | 1.761** | 1.400** |
|  | (0.100) | (0.119) | (0.177) | (0.196) |
|  |  |  |  |  |
| Observations | 1,349 | 1,349 | 703 | 703 |
| R-squared | 0.178 | 0.253 | 0.183 | 0.252 |

Standard errors in parentheses

** p<0.01, * p<0.05 for one tailed tests.

**Supplementary Information References**

Druckman, James N., and Matthew Levendusky. 2019. “What Do We Measure When We Measure Affective Polarization?” *Public Opinion Quarterly* 83(1): 114-22.

[Flores, Andrew R.,](https://williamsinstitute.law.ucla.edu/experts/andrew-r-flores/) [Jody L. Herman,](https://williamsinstitute.law.ucla.edu/experts/jody-l-herman/) Gary J. Gates, Taylor N. T. Brown. 2016. “How Many Adults Identify as Transgender in the United States?” Los Angeles, CA: The Williams Institute.

Howat, Adam J. 2019. “The Role of Value Perceptions in Intergroup Conflict and Cooperation.” *Politics, Groups, and Identities*. <https://doi.org/10.1080/21565503.2019.1629320>

Malhotra, Neil and Alexander Kuo. 2008. “Attributing Blame: The Public’s Response to Hurricane Katrina.” *Journal of Politics* 70(1): 120-135.

1. In this initial survey, 546 respondents were randomly assigned to a condition that did not measure affective polarization. Another 151 did not answer the affective polarization measure. These respondents were thus not relevant for the follow-up experiment. (Thus, the total in that survey was 4,042.) Also, this survey itself consisted of three distinct waves for reasons unrelated to this project. Also, the affective polarization measures in the survey varied the target such that some answered the conventional items asking about the Democratic and Republican parties, while others were asked about partisans who varied in terms of the amount they discussed politics (rarely, occasionally, frequently) and/or their ideology (liberal, moderate, conservative). These variations do not affect the results we present here. That is, when we include variables for the experimental conditions they do not change our findings. [↑](#footnote-ref-1)
2. Response to the COVID-19 wave is correlated with various respondent characteristics (e.g., higher income, older age, political interest) but we maintain considerable variance on those characteristics and thus are confident in our experimental in inferences. [↑](#footnote-ref-2)
3. The U.S. Census Bureau does not currently ask about transgender identity, so there is no government-provided benchmark for that quantity. Flores et al. (2016) estimate that less than 1 percent of Americans identify as transgender, consistent with our estimates here; see <http://bit.ly/2Nj5DZE> for more details. [↑](#footnote-ref-3)
4. We have a variety of other outcomes variables in our original data, but here we focus on the core set of items for our study, which are the items measuring affective polarization. [↑](#footnote-ref-4)
5. The next four items measure partisanship as a social identity; we create a measure by taking the average score ($\alpha=0.89$). [↑](#footnote-ref-5)
6. Recoded on a 0-1 scale. Here, $OUTGROUP corresponds to the other party. Here, we combine all of the affective polarization items asked here—feeling thermometer ratings, trait ratings, trust, and social distance measures—into an aggregate scale ($\alpha=0.88$). We use out-party ratings because those capture the core of affective polarization (Druckman and Levendusky 2019). In the 2019 wave, we included an experiment that varied how the parties were described in terms of their ideology and political interest (more details are available from the authors). Here, we ignore that variation, as it only adds noise to our data. We can also control for it to ensure that it does not bias our results. [↑](#footnote-ref-6)
7. The negative traits (hypocritical, selfish and mean) are reverse coded. [↑](#footnote-ref-7)
8. Reverse coded. [↑](#footnote-ref-8)
9. We recognize on this item we use “Trump administration” rather than President Trump as on the other items. We piloted both versions of the question and found the wording makes no difference. We opted for the administration wording on this item as it is more consistent with confidence in institutions items used in other surveys (e.g., the General Social Survey) whereas the other two question are more akin to personal attribution questions that name individuals (e.g., Malhotra and Kuo 2008). [↑](#footnote-ref-9)
